# 535. Comparative Evaluation of Burn Patient Skin Cell Solution: Manual versus Automated Autologous Cell Preparation

**DOI:** 10.1093/jbcr/irag033.232

**Published:** 2026-04-06

**Authors:** Sigrid Blome-Eberwein, Faith Cook

**Affiliations:** Lehigh Valley Health Network Regional Burn Center, Allentown, PA; Lehigh Valley Health Network Regional Burn Center, Easton, PA

## Abstract

**Introduction:**

Skin cell suspension autograft (SCSA) is increasingly used in acute burn and wound care to support healing while minimizing donor site requirements. Prepared from a small autologous skin sample, SCSA contains keratinocytes, fibroblasts, and melanocytes essential for epidermal regeneration. To promote consistency in preparation, an Automated Autologous Cell Harvesting Device (A-ACHD) was developed to standardize enzymatic and mechanical disaggregation. The biological attributes of A-ACHD–prepared SCSA have not been evaluated using burn patient skin samples. This study compared SCSA prepared using A-ACHD and the manual ACHD with paired skin samples from burn patients.

**Methods:**

Discarded donor skin from patients undergoing split-thickness grafting was processed with ACHD and A-ACHD per manufacturer protocols. Cell yields were quantified by propidium iodide assay and flow cytometry, with standardized gating to exclude debris. Phenotyping used antibodies for keratinocytes (pan-cytokeratin), fibroblasts (Vimentin), and melanocytes (P-mel). Non-inferiority was evaluated using a 70% margin, and paired t-tests were performed.

**Results:**

Paired donor samples from 17 patients were analyzed. Non-inferiority testing confirmed that A-ACHD–prepared suspensions were non-inferior to those generated using ACHD (total cell yield, live cell yield, percent keratinocytes, fibroblasts, and melanocytes). A statistically significant higher average total yield of cells and live yield of cells were found in suspensions prepared using the automated device (*p*=.013 and *p*=.002, respectively).

**Conclusions:**

Both devices produced viable SCSA suspensions with yields observed comparable with previously reported literature. The automated system additionally provided higher yields while standardizing preparation, supporting its role as an effective evolution of this technology.

**Applicability of Research to Practice:**

Automation of SCSA preparation constitutes a technological advancement, offering standardized processes and streamlined workflow integration that expand its accessibility for acute wound management.

**Funding for the study:**

Foundation Funding; Devices and reagents were supplied by the manufacturer.

